# Supplementary material for: Synthesis of Polystyrene-Based Cationic Nanomaterials with Pro-Oxidant Cytotoxic Activity on Etoposide-Resistant Neuroblastoma Cells
Source: Nanomaterials (Basel). 2021 Apr 10;11(4):977. doi: 10.3390/nano11040977 (PMC8069339; doi:10.3390/nano11040977)
Supplement: Supplementary file 1 [file nanomaterials-11-00977-s001.pdf]

nanomaterials

Supplementary Information

Synthesis of Polystyrene-Based Cationic Nanomaterials with Pro-Oxidant Cytotoxic Activity on Etoposide-Resistant Neuroblastoma Cells

Silvana Alfei [1,*,†], Barbara Marengo [2,†], Giulia Elda Valenti [2] and Cinzia Domenicotti [2]

[1] Department of Pharmacy, University of Genoa, Viale Cembrano, 16148 Genoa, Italy
[2] Department of Experimental Medicine (DIMES), University of Genova, Via Alberti L.B., 16132 Genoa, Italy; barbara.marengo@unige.it (B.M.); giuliaelda.valenti@edu.unige.it (G.E.V.); cinzia.domenicotti@unige.it (C.D.)
* Correspondence: alfei@difar.unige.it; Tel.: +39-010-355-2296
† These authors contributed equally to the work.

Section S1. Synthesis of 4-(4-amminobuthyl)styrene hydrochloride M5 (**5**) [1]

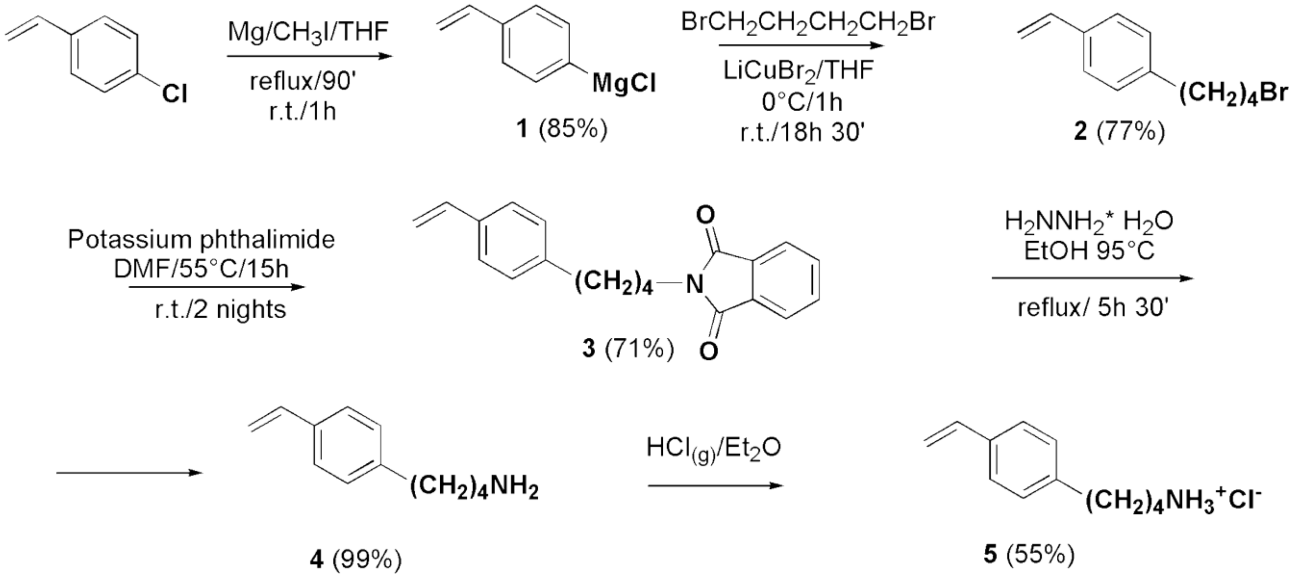

Scheme S1. Synthetic path to prepare monomer M5 (**5**).

S1.1. *p*-Vinylphenylmagnesium Chloride (**1**)

Magnesium (0.9680 g, 39.8 mmol) previously washed with anhydrous diethyl ether (Et_2O) (10 mL), anhydrous tetrahydrofuran (THF) (5 mL) and a few goggles of methyl iodide (MeI) and 4-chlorostyrene were introduced into a 100 ml three-necked flask flamed under nitrogen equipped with mechanical stirrer, condenser, dropping funnel. After starting the reaction by overheating with flame, the mixture, kept under reflux, was added with a solution of 4-chlorostyrene (5.20 g, 37.5 mmol, 4.5 mL) in THF_{dry} (50 mL) by slow dripping. After further reflux by stirring and

disappearance of the metallic magnesium (90′), stirring was continued at r.t. for 1 h. Then, the suspension was decanted to obtain a clear solution of Grignard's reagent (**1**), which will be used as it is in the subsequent reaction. To know the exact title of reagent **1**, an aliquot of the obtained solution (1 mL) was transferred in a flask containing 0.1014 N HCl in excess (10 mL) and back titrated with 0.1021 N NaOH (5.20 mL) in the presence of phenolphthalein as indicator. Reagent **1** resulted 0.580 N (85 % yield).

S1.2. 4-(4-Bromobuthyl)styrene (**2**)

A mixture of the 1,4-dibromobuthane (24.72 g, 114.5 mmol), dry THF (50 mL) and a solution of $LiCuBr_2$ in dry THF (3.4 mL) was cooled to 0 °C, treated dropwise with 0.580 N 4-vinylphenyl magnesium chloride (47 mL, 27.3 mmol) in THF and stirred at room temperature for 18 h. The reaction mixture was then treated with an iced aqueous solution of NaCN (1.81 g) and NH_4Cl (11.34 g) dissolved in water (70 mL) and extracted with peroxide-free ethyl ether (3x60 mL). The extracts were dried over anhydrous $MgSO_4$ overnight and the solvent was removed by evaporation at reduced pressure obtaining a pale yellow oil (23.72 g). The unreacted 1,4-dibromobuthane was removed by distillation at reduced pressure (0.05 torr) and the oily yellow residue was furtherly purified by chromatographic column (petroleum ether 40-60°C/acetone = 85/15) to provide **2** as colorless oil (5.01 g, 21.0 mmoli, 77 % yield).

Bp. 100 °C/0.15 torr, (lit. [2]: 92–93 °C/0.1 torr). FTIR (film, ν cm^{-1}) 990, 906 (CH_2=CH). ^1H NMR ($CDCl_3$, 300 MHz, δ ppm) 1.76 (m, 2H); 1.86 (m, 2H); 2.61 (t, 2H, J = 7.4 Hz); 3.39 (t, 2H, J = 6.6 Hz); 5.19 (dd, 1H, J_1 = 1.0 Hz, J_{cis} = 10.9 Hz); 5.70 (dd, 1H, J_1 = 1.0 Hz, J_{trans} = 17.6 Hz); 6.68 (dd, 1H, J_{cis} = 10.9 Hz, J_{trans} = 17.6 Hz); 7.11–7.34 (m, 4H). ^{13}C NMR (75.5 MHz, δ ppm) 29.74; 32.18; 33.60; 34.66; 113.05; 126.24; 128.54; 135.36; 136.61; 141.50. GC-MS (EI, m/z, %): 240 (M^+ [^{81}Br], 37); 238 (M^+ [^{79}Br], 34); 117 (100).

S1.3. *N*-[(4-Vinylphenyl)buthyl]phthalimide (**3**)

A mixture of **2** (1.17 g, 4.9 mmol), potassium phthalimide (0.9367 g, 5.1 mmol) and dry DMF (6 mL) was heated at 55 °C under nitrogen and mechanical stirring for two nights. The reagent disappearance was confirmed by TLC (toluene 100%). After removal of the solvent at reduced pressure the white solid residue was taken with chloroform (32 mL), filtered and washed with chloroform (3 x 10 mL). All the organic extracts were combined, washed with 0.2137 N NaOH (10 mL, pH = 14), water (2 x 5 mL, pH = 7-8) and dried over anhydrous $MgSO_4$. The removal of the solvent at reduced pressure afforded **3** as a crude solid (1.22 g) which was furtherly purified by chromatographic column using toluene and then toluene 100 % as eluents obtaining a white solid (1.05 g, 3.5 mmoli, 71 % yield). Mp. 114–115 °C. Purity 98 % by HPLC. IR (KBr, ν cm^{-1}) 1703 (C=O), 992, 913 (CH_2=CH). ^1H NMR ($CDCl_3$, 300 MHz, δ ppm) 1.69 (m, 4H); 2.64 (t, 2H, J = 7.0 Hz); 3.71 (t, 2H, J = 7.0 Hz); 5.18 (dd, 1H, J_{gem} = 1.0 Hz; J_{cis} = 10.9 Hz); 5.69 (dd, 1H, J_{gem} = 1.0 Hz; J_{trans} = 17.6 Hz); 6.68 (dd, 1H, J_{cis} = 10.9 Hz; J_{trans} = 17.6 Hz); 7.13–7.36 (m, 4H); 7.67–7.85 (m, 4H). ^{13}C NMR (75.5 MHz, δ ppm) 28.14, 28.53, 35.07, 37.76, 112.94, 123.18, 126.20, 128.59, 132.14, 133.87, 135.27, 136.66, 141.73, 168.43. GC-MS (EI, m/z, %): 305 (M^+, 100). Anal. Calcd. For $C_{20}H_{19}NO_2$: C, 78.66; H, 6.27; N, 4.59. Found: C, 78.62; H, 6.26; N, 4.58.

S1.4. 4-(4-Amminobuthyl)styrene (**4**)

N-[(4-Vinylphenyl)buthyl]phthalimide (**3**) (3.05 g, 10.0 mmol) was dissolved in 95% ethanol (18 mL) and treated under nitrogen and stirring at reflux with a solution of hydrazine hydrate (0.72 g, 14.4 mmol, 0.7 mL) in 95% ethanol (2 mL) for 5 h up to the disappearance of **3** (TLC, eluent toluene). After removal of the solvent at reduced pressure, the solid residue was taken with chloroform (50 mL) and treated with 20 % aqueous NaOH (30 mL). The aqueous phase was separated, extracted with chloroform (5 x 30 mL) and the extracts combined and dried over $MgSO_4$. The

removal of chloroform afforded the free base as oil (1.73 g, 9.9 mmoli, 99 % yield) which was transformed into its hydrochloride (following section 2.2.5.) without distillation. IR (KBr, ν cm^{-1}) 3302 (NH), 992, 904 (CH_2=CH). ^{1}H NMR ($CDCl_3$, 300 MHz, δ ppm) 1.16 (bs, 2H, NH_2); 1.46 (m, 2H); 1.62 (m, 2H); 2.60 (t, 2H, *J* = 7.4 Hz); 2.68 (t, 2H, *J* = 7.0 Hz); 5.17 (dd, 1H, J_{gem} = 1.0 Hz; J_{cis} = 10.9 Hz); 5.69 (dd, 1H, J_{gem} = 1.0 Hz; J_{trans} = 17.6 Hz); 6.68 (dd, 1H, J_{cis} = 10.9 Hz; J_{trans} = 17.6 Hz); 7.10–7.32 (m, 4H).

S1.5. 4-(4-Amminobuthyl)styrene hydrochloride M5 (**5**)

A solution of amine **4** (1.73 g, 9.9 mmol) in dry diethyl ether (200 mL) was cooled to 0 °C and treated under stirring up to saturation with dry gaseous hydrochloric acid (30′). The white precipitate was filtered, washed with fresh ether (50 mL), dried to constant weight obtaining a white solid (2.21 g) which was crystallized twice from 200 mL of acetonitrile (ACN) to afford the hydrochloride derivative **5** (namely M5) as flaky white solid (1.15 g, 5.42 mmoli, 55 % yield). Mp 198–200 °C (acetonitrile). IR (KBr, ν cm^{-1}) 3435 (NH_3^+), 991 and 911 (CH_2=CH). ^{1}H NMR (CD_3OD, 300 MHz, δ ppm) 1.63–1.75 (m, 4H); 2.57–2.69 (m, 2H); 2.90–2.95 (m, 2H); 5.16 (dd, 1H, J_{gem} = 1.1 Hz; J_{cis} = 10.9 Hz); 5.71 (dd, 1H, J_{gem} = 1.1 Hz; J_{trans} = 17.6 Hz); 6.69 (dd, 1H, J_{cis} = 10.9 Hz; J_{trans} = 17.6 Hz); 7.10–7.36 (m, 4H). ^{13}C NMR (75.5 MHz, δ ppm) 28.08, 29.16, 35.89, 40.70, 113.26, 117.32, 129.70, 136.97, 137.99, 142.63. Anal. Calcd. for $C_{12}H_{18}ClN$: C, 68.07; H, 8.57; N, 6.62; Cl, 16.74. Found: C, 68.05; H, 8.58; N, 6.64; Cl, 16.78.

Section S2. Preparation and Characterization of Nanoparticulate Copolymer P5 [1]

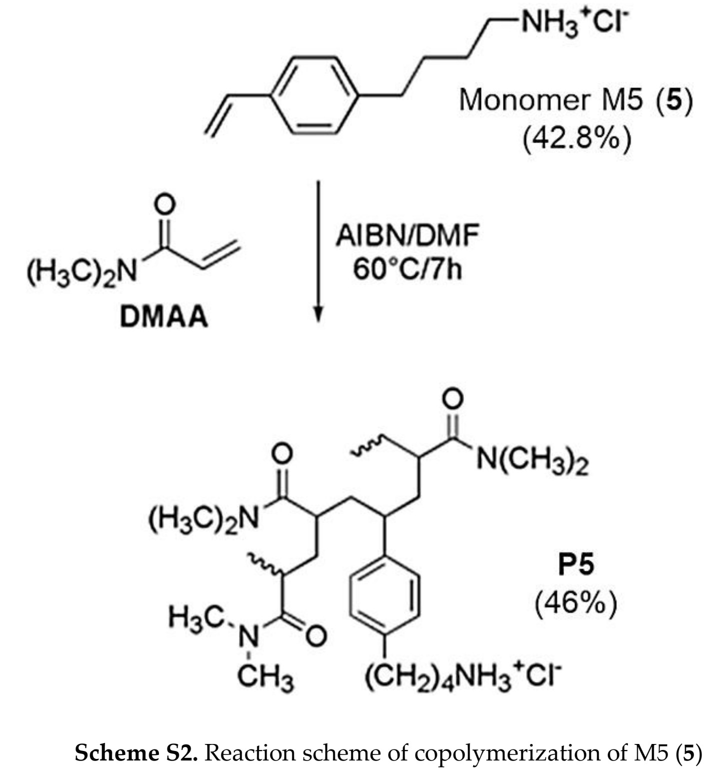

Scheme S2. Reaction scheme of copolymerization of M5 (**5**) to obtain P5.

The experimental data of the copolymerization have been reported in Table S1.

Table S1. Experimental data of copolymerization of M5 to afford P5.

M5 (mg, mmol, %[1])	DMAA (mg, mmol)	DMF (mL)	AIBN (mg, %[2])	Time (h)	P5 (g, %[3])
697.7, 3.30, 42.8	765.9, 7.7	6.5	15.3, 1.0	7	0.6677, 46

[1] loading (%) of cationic monomer; [2] percentage wt/wt (M5 + DMAA); [3] percentage of conversion.

S2.1. Copolymerization Procedure

In a 25 mL tailed test tube equipped with a magnetic stirrer and carefully flamed under nitrogen, monomer M5 (**5**) DMAA, AIBN as radical initiator, and the freshly distilled anhydrous solvent were introduced in the ratios reported Table S1. The mixture thus obtained was subjected to three vacuum-nitrogen cycles to remove the oxygen. The clear solution was then siphoned into a 25 mL flask with screw cap and silicone septum. Nitrogen was then bubbled for 5 minutes in the solution, which was subsequently left under stirring at 60 °C. The final yellow solution was evaporated at reduced pressure achieving the crude co-polymer which was subjected to three cycles of dissolution in MeOH and precipitation in Et_2O obtaining P5 as white solid. P5 was subsequently subjected to fractioning as described in the following section S2.2.

S2.1.1. Fractioning of P5

A solution of P5 in just enough MeOH was filtered and transferred in a three-necks round-bottomed flask equipped with a mechanic stirrer and a funnel. It was thermostated at 25 ° C and the clear solution (S1) was slowly added with Et_2O until an oily precipitate (OP5-1) was obtained. OP5-1 was decanted and separated from the supernatant (S2).

S2 was treated as the starting solution (S1) obtaining a second oily precipitate (OP5-2). OP5-1 and OP5-2 were then dissolved in MeOH and precipitated in an excess of Et_2O obtaining the corresponding copolymers, namely P5-High and P5-Low.

FTIR (KBr, ν cm^{-1}) 3500 (NH_3^+); 1649 (C=O); 755 (*o*-disubstituted phenyl ring).

The unreacted M5 was recovered from the mixture of the combined solvents by evaporation at reduced pressure.

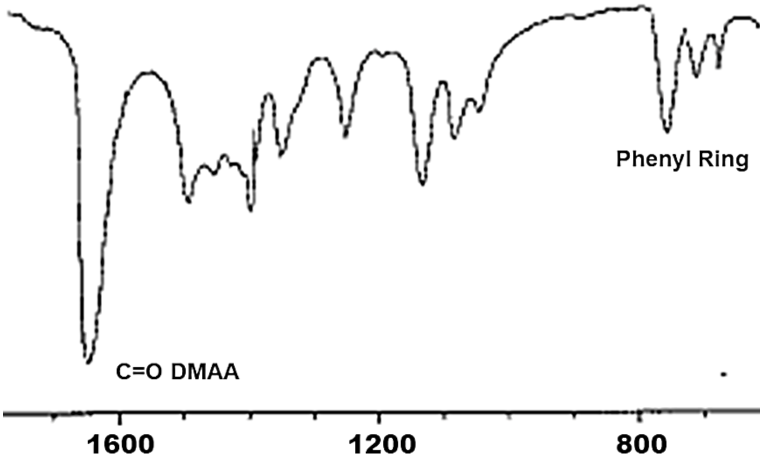

Figure S1. Significant part of FTIR spectrum of P5.

S2.2. Determination of the Average Molecular Mass (Mn) of Copolymer P5

S2.2.1. Calibration

Solutions of polyoxyethylene (PEO) with Mn 10800 in methanol (MeOH) were prepared at three different concentrations [c (mol/Kg)] and were analyzed by the vapor pressure osmometer (VPO) technique at 45 °C. The quotients of measured values (MV) and the corresponding concentrations, i.e. MV/c (Kg/mol), were determined. These data (Table S2) were used to obtain a linear regression curve according to the Ordinary Least Squares (OLS) method, whose equation was Eq. (1) and extrapolating it to concentration c = 0, K_{cal} was determined. K_{cal} was found to be 501.

$$y = 73442x + 500.92 \tag{1}$$

S2.2.2. Measurements

Solutions of P5 in MeOH were prepared at three different concentrations c (g/Kg) (Table S2) and were analyzed by VPO method at 45 °C in MeOH. The ratios between the measurement values (MV) and concentrations (c) (kg/g) were plotted *vs* concentrations (c) finding a regression curve whose extrapolation to concentration c = 0, worked directly by the instrument, provided the K_{meas} (kg/g) for P5. The molecular mass of P5 was determined accordingly to equation Eq. (2) and was reported in Table S2

$$MW\left(\frac{g}{mol}\right) = \frac{Kcal}{Kmeas} \tag{2}$$

Table S2. Data of calibration, results of measurements and estimation of Mn of P5 according to Eq. (2).

Calibration		Measurements	
c (mol/Kg)^{PEO}	MV/c (Kg/mol)^{PEO}	c (g/Kg)^{P5}	Mn (g/mol)
0.0048410	856	2.1949	
0.0058304	930	5.6195	5100
0.0068966	1007	7.7859	
K_{cal} (Kg/mol) = 501		K_{meas} (Kg/g) = 0.0982	

S2.3. Determination of NH_2 equivalents contented in P5

The NH_2 content of P5, in the form of hydrochloride, was obtained by volumetric titrations with a solution of $HClO_4$ in acetic acid (AcOH), using quinaldine red as indicator [3]. Briefly, acetic anhydride (3 mL) was added to a solution of $HClO_4$ 70% (1.4 mL) in AcOH (80 mL), obtaining a colorless solution which was left under stirring at room temperature overnight. The clear yellow solution was made up to 100 mL with AcOH and standardized with potassium acid phthalate. The title of solution was found to be 0.1612 N. A sample of P5 (300.5 mg) was dissolved in AcOH (5 mL), treated with 2 mL of a solution of mercury acetate (1.5 g) in AcOH (25 mL), added with a few drops of a solution of quinaldine red (100 mg) in AcOH (25 mL) and titrated with the standardized solution of $HClO_4$ in AcOH, using a calibrated burette with needle valve (0.02 mL). The very sharp end points were detected by observing the disappearance of the red color. Standardization and titrations were made in triplicate and the result was reported as means ± SD and expressed both as μequiv. NH_2/μmol P5 and μequiv. NH_2/g P5 (Table S3).

S2.4. Dynamic Light Scattering (DLS) analysis

The hydrodynamic size (diameter) (Z-AVE, nm) and PDI of P5 particles were determined using Dynamic Light Scattering (DLS) analysis. Z-Ave and PDI measurements were performed in water mQ as medium at max concentration of P5 of 3 mg/mL (pH = 7.4), in batch mode using a low volume quartz cuvette (pathlength, 10 mm). The analysis was performed by a photon correlation spectroscopy (PCS) assembly, equipped with a 50 mW He-Ne laser (532 nm) and thermo-regulated at the physiological temperature of 37 °C. The scattering angle was fixed at 90°. Results were the combination of three 10-min runs for a total accumulation correlation function (ACF) time of 30 min. The hydrodynamic particle size result was volume-weighted and reported as the mean of three measurements ± SD (Table S3). PDI value was reported as the mean of three measurements ± SD made by the instrument on the sample. The Z-potential (ζ-p) was measured at 37° C in mQ water as a medium, and an applied voltage of 100 V was used. The P5 sample was loaded into pre-rinsed folded capillary cells, and twelve measurements were performed.

Table S3. Results of volumetric titration of an exactly weighted amount of P5. Hydrodynamic size (nm), ζ-p and PDI at 37 °C of P5 by DLS analysis (degree of freedom = 3).

**P5 (5100)**[1] mg (mmol)	**$HClO_4$ 0.1612 N** (mL)	**NH_2** (mmol)	**μequiv.$_{NH2}$/g$_{P5}$**	**μequiv.$_{NH2}$/μmol$_{P5}$**
300.5 (0.0589)	1.67	0.2686	894	4.6
**mg/mL**	**Z-AVE Size (nm)**	**ζ-p(mV)**	**PDI**	
3	334 ± 27	+57.6 ± 1.7	1.012 ± 0.007	

[1] Mn of P5.

Section S3. Correlation between ROS production and Cell viability reduction.

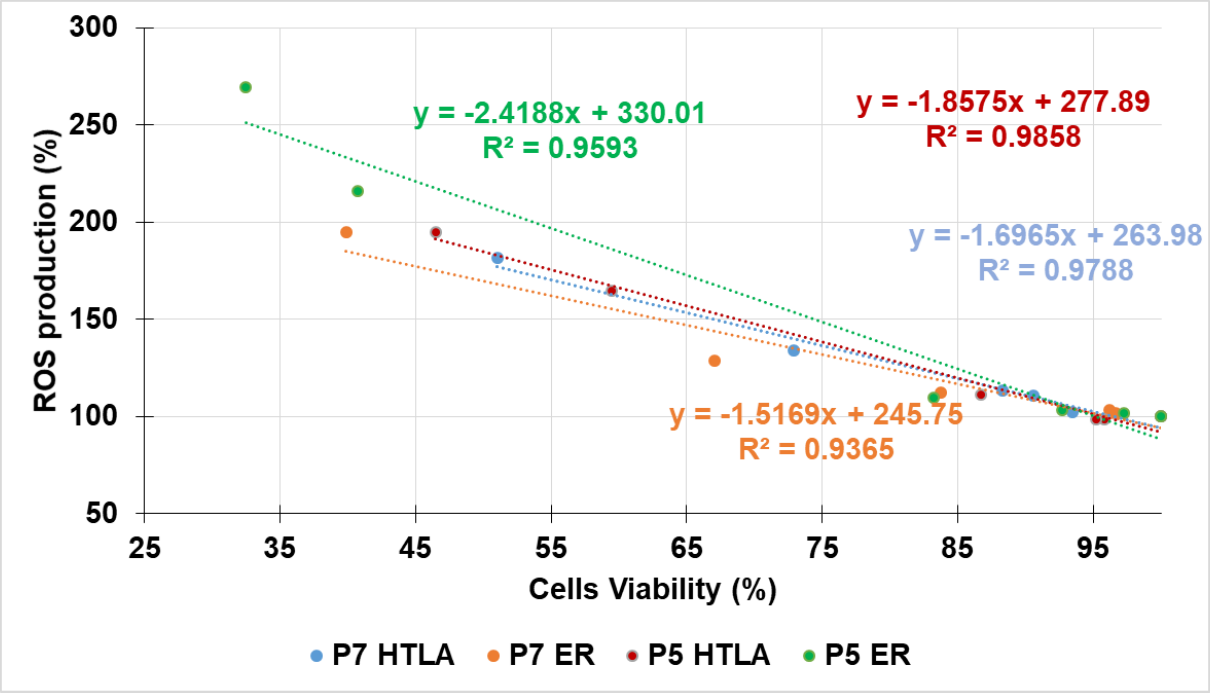

Graph S1. ROS production (%) vs cells viability (%).

References

1. Alfei, S.; Piatti, G.; Caviglia, D.; Schito, A.M.Synthesis, Characterization and Antibacterial Activity of a 4-Ammoniumbuthylstyrene-based Random Copolymer. *Polymers*, **2021**. Accepted.
2. Hirao, A.; Hayashi, M.; Nakahama, S. Synthesis of End-Functionalized Polymer by Means of Anionic Living Polymerization. 6. Synthesis of Well-Defined Polystyrenes and Polyisoprenes with 4-Vinylphenyl End Group *Macromolecules* **1996**, *29*, 3353–3358.
3. Alfei, S.; Castellaro, S. Synthesis and Characterization of Polyester-Based Dendrimers Containing Peripheral Arginine or Mixed Amino Acids as Potential Vectors for Gene and Drug Delivery. *Macromol. Res.* **2017**, *25*, 1172–1186.
